# Analysis of sex and gender-specific research reveals a common increase in publications and marked differences between disciplines

**DOI:** 10.1186/1741-7015-8-70

**Published:** 2010-11-10

**Authors:** Sabine Oertelt-Prigione, Roza Parol, Stephan Krohn, Robert Preißner, Vera Regitz-Zagrosek

**Affiliations:** 1 Institute of Gender in Medicine, Charité-Universitätsmedizin, Berlin, Germany; 2 Structural Bioinformatics Group, Dep. Of Physiology, Charité - Universitätsmedizin, Berlin, Germany; 3 Center for Cardiovascular Research (CCR), Charité-Universitätsmedizin, and German Heart Institute, Berlin, Germany

## Abstract

**Background:**

The incorporation of sex and gender-specific analysis in medical research is increasing due to pressure from public agencies, funding bodies, and the clinical and research community. However, generations of knowledge and publication trends in this discipline are currently spread over distinct specialties and are difficult to analyze comparatively.

**Methods:**

Using a text-mining approach, we have analysed sex and gender aspects in research within nine clinical subspecialties - Cardiology, Pulmonology, Nephrology, Endocrinology, Gastroenterology, Haematology, Oncology, Rheumatology, Neurology - using six paradigmatic diseases in each one. Articles have been classified into five pre-determined research categories - Epidemiology, Pathophysiology, Clinical research, Management and Outcomes. Additional information has been collected on the type of study (human/animal) and the number of subjects included. Of the 8,836 articles initially retrieved, 3,466 (39%) included sex and gender-specific research and have been further analysed.

**Results:**

Literature incorporating sex/gender analysis increased over time and displays a stronger trend if compared to overall publication increase. All disciplines, but cardiology (22%), demonstrated an underrepresentation of research about gender differences in management, which ranges from 3 to 14%. While the use of animal models for identification of sex differences in basic research varies greatly among disciplines, studies involving human subjects are frequently conducted in large cohorts with more than 1,000 patients (24% of all human studies).

**Conclusions:**

Heterogeneity characterizes sex and gender-specific research. Although large cohorts are often analysed, sex and gender differences in clinical management are insufficiently investigated leading to potential inequalities in health provision and outcomes.

## Background

Gender medicine has evolved as an independent research field over the last decades. The establishment of its scientific validity through rigorous research has been a fruitful approach which is leading to its acceptance and the growth of scientific knowledge. Gender medicine originated from specialty-specific, initially mostly cardiologic, research questions and analysis has been frequently performed in the context of a particular discipline. Research on sex and gender differences includes a range of different approaches, from basic science and etiopathogenetic research, to the analysis of differences in signs and symptoms, diagnostics and management [1-7].

Research publications in medicine increased over time and this might prompt the assumption that relevant and novel research topics are more frequently investigated today. However, this might not be true. Topics can be abandoned as time passes and interests within the scientific community shift [8]. Funding agencies are encouraging the incorporation of sex and gender aspects into biomedical research and leading scientific journals are requesting its inclusion into data analysis [9-13]. This is promoting increasing attention to the subject; however, this methodological shift appears to be a lengthy process. In fact, the expected increase in gender-specific research publications over time has never been quantified.

In addition to publication trend differences, it is not known whether some disciplines are more inclined to one research approach rather than to others, and if differences in research design and cohort size exist. Specialty-specific interests have driven the development of the discipline and might be influencing its evolution.

At present, a quantitative and exploratory analysis of gender research is lacking. An overview about the discipline's development would, however, allow for a more systematic approach to present and future research definitions. Comparison among specialties might offer insight into distinct research practice and highlight fundamental gaps that might be subsequently explored. Furthermore, information about sex differences in a specific area of medicine could be relevant to others, as basic mechanisms and differences in clinical management may be similar. Knowing that one specialty offers abundant information about, for example, pathophysiologic research could encourage exploration of similar mechanisms in related disciplines using appropriately modified approaches.

The present study offers a systematic classification of research in gender medicine to provide an initial overview of the field, highlight relevant differences in research approaches between clinical disciplines and identify relevant information gaps.

## Methods

### Selection procedure

We were interested in the identification of medical literature containing the following information:

1. research conducted in both females and males

2. description of the differences between the sexes/genders

3. presentation of sex/gender specific analysis

### Article retrieval

A specific search tool was developed for the present project in collaboration with the Bioinformatics department of our university. A text-mining algorithm (GenderMedST) based on the Lucene platform [14] and an appropriately designed database (GenderMedDB) have been used for collection and archiving of relevant literature. Apache Lucene is an open-source text-mining platform written in Java. It enables the identification of selected query terms defined by the operator in diverse text sources.

A series of search terms was defined to identify relevant literature. These were "*sex difference(s)*", "*sexual difference(s)*", "*gender difference(s)*", "*sexually dimorph(ic)*", "*sexual dimorphism*", "*sex dependent*", "*sex based*", "*gender based*", and "*gender dependent*". MeSH vocabulary was used for disease definitions and synonyms. MeSH (medical subject headings) represents the National Library of Medicine's vocabulary thesaurus. It is used for the indexing of articles within PubMed and can be applied for the identification of synonyms of disease descriptions.

Abstracts are screened by two independent graders and classified according to the following selection criteria:

Inclusion criteria:

a) Description of "sex/gender" specific differences in the analysed species (human, mouse, rat, and so on.)

b) Analysis of data with respect to sex/gender-specific differences.

Exclusion criteria:

a) Absence of sex/gender-specific description and analysis of results

b) Presence of generalized statements without descriptions of performed analysis, for example, "no gender differences were found".

c) Reference to the analysed condition (for example, "hypertension") only as co-morbidity, confounder, or anamnestic finding.

### Selection of specialties and diseases

We selected nine subspecialties in the area of Internal Medicine. The selected specialties were chosen according to standard division of clinical practice and for their ability to represent a broad range of potentially distinct approaches. The following specialties were included: cardiology, pulmonology, nephrology, gastroenterology/hepatology, rheumatology, endocrinology, neurology, haematology and oncology.

Six representative conditions were chosen within each specialty. Diseases were selected based on prevalence and previous knowledge about sex/gender differences. Specialists in the single fields were asked for input and critical evaluation of the selected diseases (Table 1).

**Table 1:** Overview of retrieved articles in each discipline

Cardiology 1,128 (2,476)	Rheumatology 146 (309)	Pulmonology 321 (566)	Nephrology 61 (171)	Gastroenterology/Hepatology 124 (305)	Neurology 434 (1,567)	Endocrinology 973 (2,771)	Oncology 135 (304)	Haematology 135 (367)
Hypertension 414 (985)	Systemic Lupus Erythematosus 68 (97)	Asthma 140 (268)	Kidney Failure 27 (99)	Hepatitis B 22 (88)	Multiple Sclerosis 65 (658)	Diabetes 447 (1,320)	Skin 45 (102)	Anemia 44 (132)
Myocardial Infarction 275 (632)	Rheumatoid Arthritis 41 (95)	Lung Carcinoma 116 (177)	Diabetic Nephropathy 11 (24)	Hepatitis C 26 (70)	Stroke 129 (334)	Obesity 349 (1,083)	Stomach 25 (63)	Leukaemia 49 (118)
Heart Failure 153 (315)	Systemic Sclerosis 3 (62)	COPD 36 (71)	Glomerulo-nephritis 9 (21)	Hepatocarcinoma 37 (53)	Alzheimer's Disease 104 (247)	Osteoporosis 123 (212)	Kidney 17 (51)	Lymphoma 34 (95)
Coronary Heart Disease 207 (386)	Fibromyalgia 15 (22)	Pulmonary Hypertension 12 (24)	Polycystic Kidney Disease 12 (20)	Inflammatory Bowel Disease 13 (41)	Epilepsy 56 (154)	Hypothyroidism 33 (80)	Bladder 22 (36)	Thrombocyto-paenia 6 (15)
Atrial Fibrillation 38 (89)	Sjögren's Syndrome 8 (18)	Pulmonary Embolism 11 (17)	Renal Artery Stenosis 0 (4)	Colon Carcinoma 24 (38)	Parkinson's Disease 69 (148)	Hyperthyroidism 16 (47)	Thyroid 16 (32)	Purpura 2 (6)
Cardiomyopathy 41 (69)	Anchilosing Spondylitis 11 (15)	Sarcoidosis 6 (9)	IgA-Nephropathy 2 (3)	AIH PBC PSC 2 (15)	Muscular Dystrophy 11 (26)	Addison/Cushing 5 (29)	Pancreas 10 (20)	Agranulo-cytosis 0 (1)

Numbers of included and (initially retrieved) articles within each discipline are provided.

For Renal Artery Stenosis and Agranulocytosis no article matched the inclusion criteria.

### Classification of literature

Literature was classified into the following five research categories: epidemiology, pathophysiology, clinical research, management and outcomes research.

Categories identified the following research:

Epidemiology - data on incidence, prevalence, mortality from survey or routine data, risk factor identification, evaluation and combination

Pathophysiology - basic research investigating pathogenic mechanisms

Clinical research - data on differences in signs and symptoms, differences in routine analyses used for clinical practice and diagnosis

Management - differences in pharmacological, invasive and non-invasive management

Outcomes - long-term consequences of the disease, its management or absence of management

Reviews were recorded separately, as this type of publication frequently contains information matching two or more of the defined categories.

### Additional data

Data on the object of study (human/animal) were extracted from the selected publications as well as the number of participants in the study as stated in the abstract. Studies employing animal models were classified as "pathophysiology".

### Publication trends of single diseases (myocardial infarction and asthma)

Publication trends of single diseases used as comparison for sex/gender specific literature were acquired through the PubMed database using the "Limits" option and identifying all publications about the investigated disease published from 1.1.year to 31.12.year.

### Statistical analysis

All information was stored in a "MySQL" (Oracle, Redwood City, CA, USA) database. MySQL is a relational database management system. Descriptive statistics were performed using the "Query Browser" tool included in the MySQL GUI programs. These additional features and upgrades to the MySQL program enable the selection and analysis of specific sets of data within the database. Numerical outputs were then transferred into Excel (Microsoft Corp., Redmond, WA, USA) spreadsheet application for graphics design.

For comparison of publication trends, log(2) of the overall numbers and of the sex/gender specific publications were calculated and tabulated. The comparison was completed by the ratio between the two values × 1,000.

## Results

### Quantification of publications

A total of 8,836 articles containing the defined search terms were identified by the text-mining tool. Of these, 3,499 matched the inclusion criteria and were further classified according to the pre-defined categories. Publication numbers varied greatly among disciplines and diseases (Table 1). Of all specialties analysed, Cardiology (n = 1,128) and Endocrinology (n = 973) contained most literature including sex/gender-specific analysis, while Nephrology (n = 171) included the least. Three conditions (hypertension, diabetes and obesity) offered more then 300 articles matching our selection criteria; two additional ones (myocardial infarction and coronary artery disease) led to 200 to 300 publication hits. However, great variability characterized these findings. Five or fewer matching publications were retrieved in six cases (renal artery stenosis, IgA nephropathy, AIH/PBC/PSC, Addison's/Cushing, purpura, agranulocytosis). Intra-specialty variations are also significant, as most disciplines include diseases with very different incorporation of sex/gender-specific analysis.

### Sex/gender specific literature increased more than disease-specific publications

**Figure 1:**
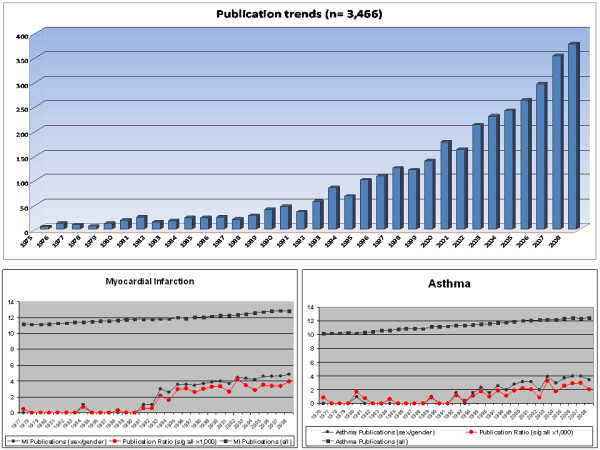
**Comparison of publication trends for general and gender-specific literature**. **(A) **Absolute numbers of gender-related yearly publications within the nine disciplines analysed are presented (n = 3,466). Publications are limited between 1975 and 1989, while they increase more markedly thereafter. **(B) **Publications trends in the field of Myocardial infarction (MI) and Asthma between 1976 to 1977 and 2008. Black squares indicate the total yearly number of publications within the field; black dots represent the number of sex/gender-specific publications within the field; red squares indicate the log(2) ratio between sex/gender literature and general literature. The noted increase after 1990 is recapitulated by these single diseases. Publications increase more in the field of gender medicine compared to overall trends (as expressed by the ratio between the trends).

A progressive increase of literature incorporating sex and gender differences appears with an almost linear progression. 1994 represented the first year where more then 50 relevant publications in different disciplines where identified; in 1997 publications reached 100. More then 350 publications from all analysed disciplines were identified in 2008 (Figure 1A). However, publication numbers have been constantly increasing since the 1980s and PubMed does now contain millions of articles. To investigate whether the trend of sex/gender-specific publications simply matched the overall expansion, we analysed two exemplary conditions: myocardial infarction and asthma. In both cases overall publications have augmented linearly as displayed in Figure 1B. Comparing gender-specific publications, however, one can see a marked increase since the 1990s with a steeper progression compared to the overall numbers. This is confirmed by the definition of the ratio between the two. Sex/gender-specific publications have, thus, been increasing more markedly than overall disease-specific publications.

### Research approaches vary within single disciplines

**Figure 2:**
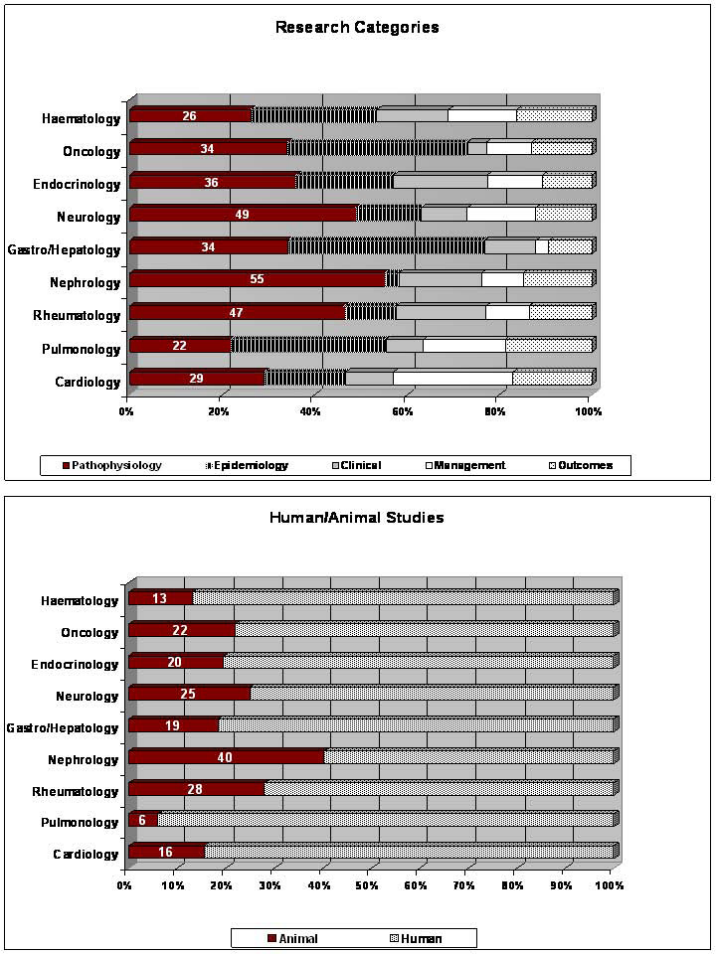
**Analysis of gender-related publications**. **(A) **Literature was classified into five research categories: Epidemiology, Pathophysiology, Clinical research, Management, and Outcomes. Research categories within the nine disciplines studied are illustrated. Equal distribution of research efforts would lead to a similar number of publications within each category. However, the categories of Pathophysiology and Epidemiology appear to be the most intensively investigated areas in most specialties. **(B) **The percentage of studies conducted on humans or animals was investigated. As all animal studies have been classified as "Pathophysiology" research, the relative percentages of animal model use can be derived by the ratio between the "Pathophysiology" data and the "Animal Study" data. Animal models are frequently used for basic research about sex differences; however, significant differences can be observed between specialties.

As quantitative differences distinguish the analysed disciplines and the diseases included in each one, we were interested in potential differences in research approaches. Analysing single disciplines as a whole offers a general overview of the distribution of different research approaches (Figure 2A); however, the single conditions might bear specificities. Neurology is an example of this phenomenon (Additional File 1; Figure S1). Pathophysiology is the most represented type of research performed and represents 42% of the total research output. This trend is recapitulated by the individually analysed conditions, but variations can be noted around this average value (stroke 20%; multiple sclerosis 69%). Clinical research (2 to 16%) is generally underrepresented as is research on outcomes (0 to 12%) with the exception of stroke (21%). Diseases leading to low numbers of sex/gender-specific publications, such as muscular dystrophy in the case of neurology (Table 1, n = 11), might not include all types of the investigated research publications.

We classified reviews as a separate category as these publications frequently encompass research data from different sources. Nonetheless, this information is significant as reviews reflect the development of scientific knowledge based on original publications. Percentages of review publications are stable ranging from 8% (Multiple Sclerosis) to 16% (Alzheimer's Disease).

### Differences in the use of animal models in basic research

Animal models represent one tool for the investigation of disease pathogenesis; however, these models are used more frequently in some fields than in others. To identify differences in utilization of animal models for sex-specific research, the appropriate data were collected and compared to the overall percentages of pathophysiology research (Figure 2B). If all basic research was performed in animal models, the two percentages should be identical. This is never the case, but more substantial variations exist. As illustrated in part A, Nephrology (55%), Neurology (49%) and Rheumatology (47%) were the disciplines with the highest frequencies of basic research. However, research on animal models represents 73% of basic research (40% of all publications) in Nephrology, 51% (25% of all publications) in Neurology and 60% (28% of all publications) in Rheumatology.

The discipline with the least amount of publications on basic research was Pulmonology (22%). Of this total, only 27% was performed in animal models (6% of all research in the field).

### Study population size in human studies

**Figure 3:**
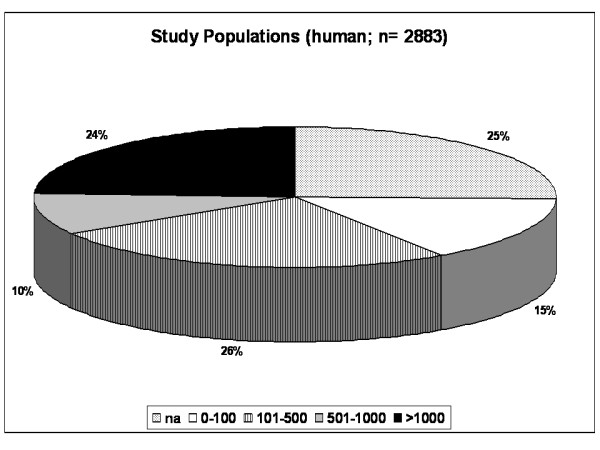
**Size of clinical studies in gender medicine**. Pie chart showing the numbers of enrolled individuals in research projects involving human subjects. 60% of the studies included 100 or more subjects. 24% of all analyses were conducted in studies involving more than 1,000 participants.

After evaluating the number of research publications involving animal models and human subjects, we were interested in the quantification of the enrolled study subjects. Most studies included 101 to 500 study subjects (26%), followed by publications about research including 1,000 or more subjects (24%; Figure 3). A total of 1,000 or more subjects was also the most frequently chosen cohort size in both Cardiology (33%) and Oncology (31%, data not shown).

### Lack of investigation of sex/gender differences in management research

**Figure 4:**
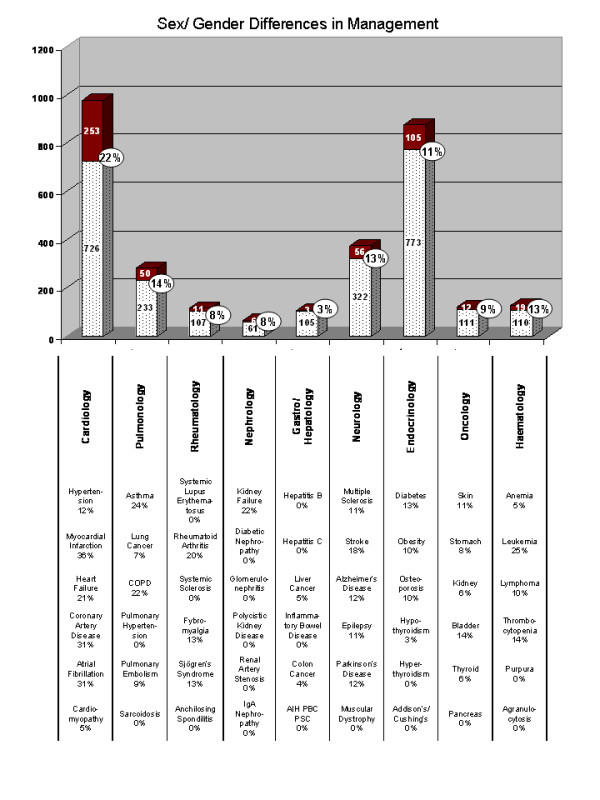
**Studies on clinical management appear underrepresented in gender medicine**. The bar graphs represent the total number of gender-related publication in each of the nine fields. Studies on clinical management are highlighted in red. The percentage of studies about management with respect to the overall number of gender-specific studies for each category is also reported as the percentage. The number of publications on clinical management within single diseases is shown below the bar graph. All disciplines, but Cardiology (22%), display low percentages of research conducted on gender differences in management.

If research was equally distributed within the five categories we used for classification, an equal distribution of 20% could be expected in each. This does not appear to be the case and imbalances affect some types of research more than others (Figure 4). Of all categories, research about sex and gender differences in management is consistently performed less than other types of research. With the exception of Cardiology (22%), the only specialty to exceed the 20% mark, in all other cases research about management differences represents 14% or less of all investigations. Gastroenterology/hepatology offers the least information about these differences (3%) and four of the six diseases analysed do not provide any information on the subject.

Not surprisingly, given the history of gender medicine, myocardial infarction (36%) and coronary artery disease (31%) offer most information on the subject, together with atrial fibrillation (31%). Asthma (24%), COPD (22%), kidney failure (22%) and leukemia (25%) also offer relatively significant amounts of information about differences in management.

## Discussion

Gender medicine is a novel and rapidly evolving discipline of research. The present study shows how in the last 30 years, publications including sex and gender differences have progressively increased and more strikingly since the 1990s. Different medical disciplines incorporate this type of analysis in different manners, choosing distinct research approaches, experimental designs and cohort sizes; however, one feature common to all disciplines, but cardiology, is the dramatic underrepresentation of investigation of sex and gender differences in clinical management.

This particular aspect needs to be emphasized and critically evaluated. Research on clinical management, that is, diagnostic approaches, referral practices, invasive and non-invasive therapy choices, is essential in understanding, shaping and improving our everyday clinical practice. Lack of knowledge about gender differences and inadequacies in health care provision have led to significant and potentially fatal imbalances in outcomes. This has been demonstrated in the field of cardiology, where the numbers of women dying of heart infarction at a young age [15-17] significantly dropped after two decades of research and the dissemination of essential information about gender differences in clinical presentation, symptoms, diagnostic and therapeutic approaches [18,19]. Examples from other disciplines are following [20,21]; however, the information is still scarce and the benefits of this additional knowledge not yet widely accepted by the medical community.

Despite this critical lack of information we found a relevant increase in sex and gender-specific analysis over time, with publications markedly rising since the 1990s. This confirms early reports from the 1990s conducted on a limited set of medical journals [22,23]. The phenomenon might be related to several factors. On the one side, the previously mentioned eye-opening reports about unexpectedly high numbers of female fatalities due to heart infarction [16] might have played a role, as well as the predominantly female fatalities related to torsade de pointes as a side effect of drug therapy [24,25]. Both events have highlighted a previously ignored problem and created the momentum for the implementation of guidelines by the FDA [12] and NIH [11,26]. Nonetheless, although the attention toward the topic is increasing, the publication numbers we are identifying are far from satisfactory.

This type of research might still be constrained by limited funds, as there are no specific funding agencies and still few calls for sex and gender-specific research. The difficulty in achieving publications in high impact journals might also play a role. While gender medicine is gaining attention in the scientific community, as demonstrated by the recent editorials in influential journals [10,27-30], several editors do not consider the topic worthy of publication and refer authors to the few gender medicine journals. Some causes for this imbalance might also be inherent in the project design. Information about sex and gender specific analysis might be included only in the body of the article and thus not captured by our research strategy; however, this should account only for a minority of the publications. Furthermore, it should be clarified that gender specific analysis is not limited to the enumeration of the number of female and male subjects in the study cohort, but extends beyond that in the form of distinct subgroup analyses [31]. This is not performed in many research publications and failure to do so may lead to incomplete or biased results leading to wrong conclusions and actions [32]. Misleading and, at times, incorrect use of the terminology "sex" and "gender" also plays a role. Authors not familiar with the subject oftentimes use the words interchangeably or use the term "gender" for any distinction between subjects, even if this is related to purely biological differences. This represents a further hurdle to the systematic analysis of publications. If one wanted to clearly identify which articles analysed solely biological differences and which ones cultural and psychosocial distinctions, many publications identifying "gender differences" would have to be reclassified as "sex differences". This warrants further analysis in the future.

Our analysis also revealed how the percentage of basic research performed in animal models varies greatly among disciplines. This is an interesting finding and may reflect intrinsic differences in the use of animal models in different specialties. The importance of using both female and male animals in basic research for etiologic investigations or drug testing is being increasingly recognized [29]. Sex differences in rodent and other animal models have been identified in wild type and congenic animals used in diverse disciplines. However, the variability of reaction in female animals due to their hormonal cycle and the potentially increased costs related to the need for larger numbers of animals to account for this variability during analysis still deters many investigators from doing so.

Last, a systematic collection of data is instrumental for research development and identification of needs and relevant questions. Diversity is the main focus of this area of research, but limited the possibility for comparison of strategies in the past. As we have seen, management research needs much attention if we strive to improve gender-sensitive health care, and although sex and gender-specific analyses are increasing, they are not increasing nearly as much as good research practice would lead us to expect. The present work represents a basic assessment of the status quo and will be used as basis for a public database, which will be made accessible to researchers in the field and open for input of their own research publications. We believe that this platform will encourage exchange among researchers interested in sex and gender-specific research and offer opportunities for fruitful cooperation to close some of the gaps identified in this analysis.

## Conclusions

The present work represents the first systematic analysis of the incorporation of sex and gender in research design and evaluation in medicine. Marked heterogeneity characterizes different disciplines, possibly reflecting research approaches and gender roles in the specialties themselves. While a progressive increase in the literature can be noted, a striking underrepresentation of research about gender differences in management characterizes all disciplines but cardiology. Given that the area of clinical management has been identified as crucial in the perpetuation of differences in health care provision, this gap needs to be addressed and possibly closed in the future. This data is also needed for the update and improvement of clinical guidelines to include information about sex and gender differences. We believe that all areas where less then 10% of the performed research is about differences in management, that is, Gastroenterology, Nephrology, Rheumatology and Oncology, definitely need to improve this area. However, most other disciplines only slightly exceed these 10% and are thus amenable to improvement as well. Specific analysis of differences in symptoms, diagnostic accuracy and therapy provision need to be addressed. To date the area of Cardiology can be considered the most productive in addressing these concerns and also in partially translating some results into recommendations for practice.

Furthermore, the use of large cohorts for human studies does not automatically guarantee gender-specific analysis and should thus be regarded as a positive evolution, but not a solution. Gender aspects have to be actively identified and analysed in each study.

## Competing interests

The authors declare that they have no competing interests.

## Authors' contributions

SOP designed the research, carried it out and wrote the manuscript. RP designed the database and performed analyses. SK collected and analysed data and reviewed the manuscript. RP supervised the database development and reviewed the manuscript. VRZ designed the research, acquired funding and reviewed the manuscript. All authors read and approved the final manuscript.

## Pre-publication history

The pre-publication history for this paper can be accessed here:

http://www.biomedcentral.com/1741-7015/8/70/prepub

## Supplementary Material

Additional file 1 **Supplementary figure 1**. **Distribution of gender literature with respect to research categories: the example of Neurology**. The field of Neurology was chosen as an example to investigate the variability in research approaches within different diseases part of the same field. **(A) **Distribution of gender-related studies according to research categories in the overall field of Neurology. **(B) **Distribution of gender studies with respect to research categories across the six different diseases chosen for the field of Neurology. The distribution trends observed in the field as a whole display variation within single diseases (see the text for details). Click here for file
