# Supplementary figures and images for: Analysis of sex and gender-specific research reveals a common increase in publications and marked differences between disciplines
Source: BMC Med. 2010 Nov 10;8:70. doi: 10.1186/1741-7015-8-70 (PMC2993643; doi:10.1186/1741-7015-8-70)

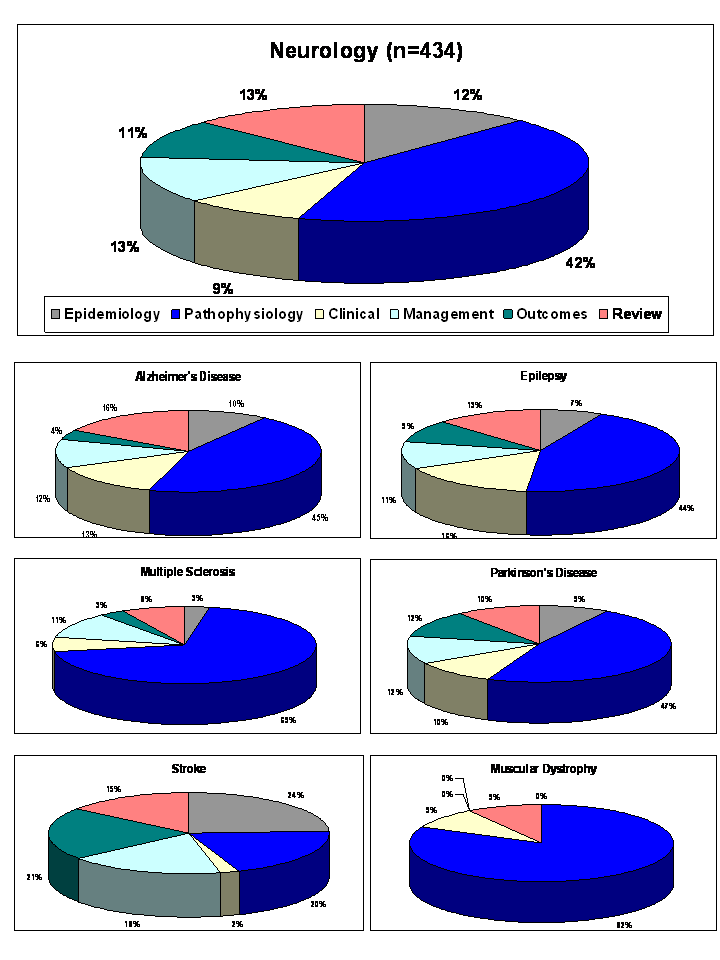

Supplement: Additional file 1 — Supplementary figure 1. Distribution of gender literature with respect to research categories: the example of Neurology. The field of Neurology was chosen as an example to investigate the variability in research approaches within different diseases part of the same field. (A) Distribution of gender-related studies according to research categories in the overall field of Neurology. (B) Distribution of gender studies with respect to research categories across the six different diseases chosen for the field of Neurology. The distribution trends observed in the field as a whole display variation within single diseases (see the text for details). [file 1741-7015-8-70-S1.TIFF]
